# Evaluation of Antibiotic Biodegradation by a Versatile and Highly Active Recombinant Laccase from the Thermoalkaliphilic Bacterium *Bacillus* sp. FNT

**DOI:** 10.3390/biom14030369

**Published:** 2024-03-19

**Authors:** Jorge Sánchez-SanMartín, Sebastián L. Márquez, Giannina Espina, Rodrigo Cortés-Antiquera, Junsong Sun, Jenny M. Blamey

**Affiliations:** 1 Facultad de Química y Biología, Universidad de Santiago de Chile, Alameda 3363, Santiago 9170022, Chile; rodrigo.cortes@usach.cl; 2 Fundación Biociencia, José Domingo Cañas 2280, Santiago 7750132, Chile; jsanchez@bioscience.cl (J.S.-S.); smarquez@bioscience.cl (S.L.M.); 3 Green Chemical Engineering Technology R&D Center, Shanghai Advanced Research Institute, Chinese Academy of Sciences, Haike Road 99, Shanghai 201210, China; sunjs@sari.ac.cn

**Keywords:** wastewater bioremediation, pharmaceutical pollutants, antibiotics, tetracyclines, fluoroquinolone, β-lactam, docking, extremozyme, oxidoreductase, spore-coat laccase

## Abstract

Laccases are industrially relevant enzymes that have gained great biotechnological importance. To date, most are of fungal and mesophilic origin; however, enzymes from extremophiles possess an even greater potential to withstand industrial conditions. In this study, we evaluate the potential of a recombinant spore-coat laccase from the thermoalkaliphilic bacterium *Bacillus* sp. FNT (FNTL) to biodegrade antibiotics from the tetracycline, β-lactams, and fluoroquinolone families. This extremozyme was previously characterized as being thermostable and highly active in a wide range of temperatures (20–90 °C) and very versatile towards several structurally different substrates, including recalcitrant environmental pollutants such as PAHs and synthetic dyes. First, molecular docking analyses were employed for initial ligand affinity screening in the modeled active site of FNTL. Then, the in silico findings were experimentally tested with four highly consumed antibiotics, representatives of each family: tetracycline, oxytetracycline, amoxicillin, and ciprofloxacin. HPLC results indicate that FNTL with help of the natural redox mediator acetosyringone, can efficiently biodegrade 91, 90, and 82% of tetracycline (0.5 mg mL^−1^) in 24 h at 40, 30, and 20 °C, respectively, with no apparent ecotoxicity of the products on *E. coli* and *B. subtilis*. These results complement our previous studies, highlighting the potential of this extremozyme for application in wastewater bioremediation.

## 1. Introduction

Water plays a vital role in the planetary ecosystem, being essential for the survival of all living organisms. In the current context of climate change emergency and global water crisis, water pollution constitutes a considerable threat to the ecosystem and human health, and when water is limited, serious environmental, health, and social problems can arise [1,2,3]. Consequently, research aimed at finding new and effective environmentally friendly methodologies for the control of polluting discharges, the treatment of contaminated wastewater, and the efficient management of water resources is a crucial task for the sustainable development and maintenance of this indispensable natural resource. 

To date, different wastewater management methods are currently used [1], however, most of these treatments are not sufficient to remove persistent organic pollutants such as synthetic dyes, polycyclic aromatic hydrocarbons (PAH), pharmaceuticals, and personal care products. Among these, antibiotics have received growing attention in recent years, as their constant discharge and spread in the environment present a high threat to public health as well as producing significant damage to the ecosystem [4]. 

Antibiotics are natural, synthetic, or semi-synthetic compounds of low molecular weight (<1000 daltons) that exert bactericidal or bacteriostatic effects. Their introduction into clinical use was possibly the greatest medical breakthrough of the 20th century [5]. Currently, there are more than 250 different registered antibiotics [6], which are classified according to their chemical structure and mechanism of action. The main groups being β-lactams, tetracyclines, macrolides, fluoroquinolones, sulfonamides, aminoglycosides, and glycopeptides antibiotics [7]. 

Global antibiotic consumption has increased immensely in recent years, and it is estimated that more than 200,000 tons are used worldwide each year [8]. They are used for the prevention and treatment of bacterial infections and diseases in humans, livestock, and aquaculture, as well as for the prevention of bacteria-induced crop damage [9]. Moreover, it has been predicted that the utilization of antibacterial drugs will continue to grow to 200% by 2030 [10]. 

One of the problems associated with this high global consumption is that substantial amounts of the antibiotics that are not metabolized pass into the environment, as conventional water treatment processes cannot effectively remove them [9,11,12]. Antibiotics are continuously discharged in aquatic environments, being found in water in the range of ng L^−1^–μg L^−1^ [13], although at point sources like hospital effluents or production sites, concentrations can even reach the high mg L^−1^ range [14]. These drugs in the ecosystem can alter microbial biodiversity, which is key for maintaining biological processes in water and soil, including biogeochemical cycles. The effects of antibiotics on ecological functions may cause changes in nitrogen transformation, methanogenesis, sulfate reduction, nutrient cycling, and degradation of organic matter, causing an ecological imbalance [13]. In addition, exposure to antibiotics may also negatively impact non-target aquatic organisms, including freshwater algae [15], zooplankton [16], crustaceans, and fishes [17], by inducing chronic effects such as changes in behavior, reproduction, and growth [11,18]. 

Furthermore, the presence of antibiotic residues presents a public health concern as it could accelerate the evolution of antibiotic-resistant bacteria (ARB) and antibiotic resistance genes (ARG) in the environment, decreasing the effectiveness of therapeutic treatments and contributing to the current global antibiotic resistance crisis [19]. The discharge of wastewater effluents into rivers, lakes, and other surface water promotes the generation and persistence of ARB and ARG [20,21,22]. These have been detected in soil, sediments, and water bodies, including wastewater, drinking water, and seawater, from different parts of the world, such as Chile [22], Uruguay [23], the United States [24], China [25], India [26], South Korea [27], Germany [28], Portugal [29], and even Antarctica [30].

Effective removal of these emerging contaminants has proven to be a major challenge using current wastewater treatment technologies [31,32]. Biological transformation of xenobiotic compounds through bioremediation processes using microorganisms or their enzymes is presented as an attractive alternative to current physical adsorption, membrane separation, electrochemical processes, and advanced oxidation methods, being an efficient and environmentally friendly approach [18,33,34,35,36]. Specifically, enzyme-mediated biotransformation is regarded as a prospective choice for the removal of pharmaceutical pollutants, presenting several advantages, including high catalytic activity, mild reaction conditions, low energy input, and a reduced amount of waste generation in comparison to traditional processes [37,38,39,40]. 

Laccases (EC 1.10.3.2, benzenediol: oxygen oxidoreductase) are versatile enzymes that belong to the protein family of multicopper oxidases and have been labeled as eco-friendly enzymes due to their ability to catalyze the one-electron oxidation of aromatic or non-aromatic compounds by reducing molecular oxygen (co-substrate) to water (as the only by-product) [41,42,43]. Moreover, the use of redox mediators alongside laccases expands the range of substrates oxidizable by these enzymes through indirect catalysis. They are suitable compounds (i.e., small diffusible electron carriers) that act as intermediate substrates for the enzyme, undergoing an oxidation–reduction cycle and constituting the laccase-mediator system (LMS), which allows the degradation of recalcitrant compounds that are not directly oxidized, either because they are too large to enter the enzyme active site or because they have a redox potential higher than the laccase itself [44]. For these reasons, laccases have attracted increasing interest for biotechnological applications, especially in bioremediation of environmental emerging and persistent environmental pollutants, including antibiotics [45,46,47]. 

Molecular docking approaches have been widely used for estimating the binding affinity between enzymes and diverse substrates, thereby facilitating the comprehension of protein–ligand interaction mechanisms. Previous investigations employing molecular docking have explored the binding of laccase enzymes with different antibiotic families, including β-lactams [8], tetracyclines [48], and fluoroquinolone [49] groups, which have provided insights into the binding modes, binding free energies, and key residues involved in the interactions between laccase enzymes and antibiotics.

The recombinant spore-coat laccase from the thermoalkaliphilic bacterium *Bacillus* sp. FNT (FNTL) has been previously characterized. It is highly active in a wide range of temperatures (20–90 °C), thermostable, and versatile, which make it a promising candidate for industrial applications. It has been shown to be capable of rapidly biodecolorizing eight recalcitrant synthetic dyes from the three most important types: azo, triarylmethane, and anthraquinone at 40 °C, using acetosyringone (AS) as a redox mediator [50], and oxidizing three PAH: anthracene, benzo[a]anthracene, and benzo[a]pyrene with the aid of 2,2’-Azino-bis (3-ethylbenzothiazoline-6-sulfonic acid) (ABTS) as redox mediator after 7 days at 30 °C [51].

This study focuses on the application of molecular docking techniques to investigate the interactions between FNTL and structurally diverse and highly consumed antibiotics from the tetracyclines, β-lactams, and fluoroquinolones families. Then, four representative antibiotics were selected for analysis of receptor–ligand interactions and to perform experimental biodegradation assays at 40, 30, and 20 °C at pH 6.0, for 24 h, with and without the addition of the natural redox mediator AS. The biodegradation of each antibiotic was quantitatively evaluated by HPLC, and the reduction in the antimicrobial effect and the ecotoxicity of the biotransformation products were qualitatively evaluated in *E. coli* and *B. subtilis.* The results not only enhance our comprehension of laccase–antibiotic interactions at the molecular level but also emphasize the relevant potential of extremozymes for various industrial and biotechnological applications, particularly in the effective wastewater bioremediation of emerging and persistent environmental pollutants.

## 2. Materials and Methods

### 2.1. Homology Modeling of Bacillus sp. FNT laccase (FNTL)

The three-dimensional homology model of FNTL was constructed using the SWISS-MODEL server [52]. The Alphafold predicted 3D structure of a “multicopper oxidase domain-containing protein” (UniProtKB ID: A0A6I7FGV2_9BACI) from *Bacillus* sp. NSP91 was used as a template for the modeling, as it was the highest ranked and highest quality template according to the SWISS-MODEL template identification pipeline. The stereochemical quality of the three-dimensional model was evaluated using Ramachandran plots and scoring functions provided by various structure validation tools, such as Prosa-web, Expasy’s QMEANDisCo, ERRAT, and Verify 3D; the latter two are available on the SAVES v6.0 server of the DOE’s Institute for Genomics and Proteomics (https://www.doe-mbi.ucla.edu/verify3d/ (accessed on 15 October 2023).

### 2.2. Preparation of Ligands and Receptors for Docking

The 3D homology model obtained with SWISS-MODEL was prepared to be used as a receptor for docking calculations. To mitigate steric hindrance in docking systems, the model underwent an energy minimization process involving 5000 steps with the GROMACS software version 2019.1 [53] using the steepest descent method and charmm36 force field. The titration states were assigned using the PDB2PQR tool [54] and applying PROPKA [55] at three distinct pH values (5.0, 6.0, and 7.0). Subsequently, the energy-minimized receptor structure underwent further processing through the prepare_receptor script from AutoDockTools4 [56]. This script facilitated the removal of all non-polar hydrogens and the generation of files in pdbqt format suitable for the docking simulations.

The chemical structures of nine antibiotics from three structurally different groups were obtained in SMILES format from the PubChem database [57]: tetracyclines group: tetracycline (TC), oxytetracycline (OTC), and doxycycline (DC); β-lactams group: ampicillin (AMP), amoxicillin (AMX), and benzylpenicillin (PEN); and fluoroquinolones group: ciprofloxacin (CIP), norfloxacin (NOR), and ofloxacin (OFX). Additionally, four different redox mediators were also included in the analysis: AS, syringaldehyde (SA), 1-hydroxybenzotriazole (HBT), and ABTS (main abbreviations used have been summarized in Table 1). Ligands were subsequently converted into 3D mol2 files and subjected to energy minimization employing the Generalized Amber Force Field (GAFF) with Open Babel [58]. The resulting energy-minimized ligand structures underwent further processing using the *prepare_ligand* script from AutoDockTools4 [56]. This script facilitated the generation of all-torsion active pdbqt files for each ligand while also incorporating polar hydrogens, considering their protonation state determined at specified pH values (5.0, 6.0, and 7.0). 

### 2.3. Molecular Docking Experiments 

To evaluate laccase–ligand affinity in terms of free energy of binding (Δ*G*_b_), ligand structures underwent docking simulations within the laccase model as a receptor using in-house wrapper scripts tailored for virtual screening with Autodock Vina [59]. The substrate-binding pocket located near the T1 copper site was identified by superimposing the receptor model on a set of *Bacillus subtillis* CotA crystals (PDB IDs: 1OF0, 7Y8B, 7Y8C, 4YVN, and 3ZDW), co-crystallized with different substrates (ABTS, syringic acid, sinapic acid, and SA) using the STAMP package for structural alignment [60]. Richard’s solvent-accessible surface area and volume of the pocket were measured using the CASTp server [61]. The center of mass of the latter pocket determined the center of the grid box for docking (53.8, 60.9, and 42.7). A cubic box measuring 27 Å along the X, Y, and Z axes was established as the grid size for the docking calculations, considering a grid space of 0.375 Å. Binding energies were computed using the empirical-based scoring function implemented in Autodock Vina with a search exhaustiveness parameter set to 32 and random seeds. Each combination of ligand–receptor–pH was subjected to ten technical replicates to ensure robustness and reliability in the experimental observations. From each docking calculation, the pose with the lowest free binding energy was selected for further analysis. The results were examined using Python Pandas data analysis functions, including the use of z-score functions for outlier removal. The results were presented as hierarchical clustered heatmaps generated using the Seaborn data visualization library. ABTS re-docking to 1OF0 laccase crystal was used as a positive control to validate the method.

### 2.4. Interaction Analysis and Visualization

The examination of protein–ligand interactions used the Protein–Ligand Interaction Fingerprints (ProLIF) tool [62]. Two-dimensional interaction diagrams were created to depict the various types of molecular interactions occurring between the amino acid residues at the binding site of FNTL and the ligands (TC, OTC, AMX, and CIP). Open-source PyMOL was used to produce all 3D visualizations and representations of the laccase model, as well as its interactions with ligands.

### 2.5. Recombinant Overexpression and Partial Purification of FNTL

*E. coli* BL21 cells (New England Biolabs, Ipswich, MA, USA) were chemically transformed using the pJ444-*fntlac* plasmid [50]. This vector contains the laccase-encoding gene (*fntlac*) from *Bacillus* sp. FNT (deposited in “Fundación Biociencia Proprietary Extremophiles Collection”) under the control of T5 promoter and a kanamycin resistance gene for selection. Transformants were grown aerobically in 400 mL TBA culture medium supplemented with 0.3 g L^−1^ CuSO_4_ and 50 μg mL^−1^ kanamycin at 23 °C with shaking at 160 rpm for 30 h [51]. The cells were harvested by centrifugation at 7690× *g* for 15 min at 4 °C, resuspended in 40 mL of lysis buffer (50 mM Tris–HCl, pH 8.0), and incubated at 37 °C for 45 min with 1 mg mL^−1^ lysozyme.

Cell disruption was achieved after three cycles of 5 min sonication using a Branson 450 Digital Sonifier (Marshall Scientific, Hampton, NH, USA), and the cell lysate was centrifuged at 17,310× *g* for 30 min at 4 °C. The cell-free extract was then heat denatured at 85 °C for 5 min using a dry bath incubator (MS Major Science, Taoyuan, Taiwan). This treatment precipitates thermosensitive native proteins from *E. coli*, allowing rapid and easy partial purification of the recombinant thermophilic FNTL. Finally, the soluble crude extract was obtained by centrifugation at 17,310× *g* for 30 min at 4 °C, and it was concentrated using an Amicon Ultra 15 (30 kDa MWCO) centrifugal filter device (Merck Millipore, Burlington, MA, USA) before utilization in antibiotic biodegradation experiments. FNTL overexpression and partial purification were evaluated using 10% sodium dodecyl sulfate polyacrylamide gel electrophoresis (SDS-PAGE) and visualized by staining with Coomassie brilliant blue R-250 [63] (Figure S1). Protein concentration was determined using the method of Bradford [64] with the Bio-Rad protein reagent (Bio-Rad Laboratories, Irvine, CA, USA) and bovine serum albumin (BSA) as the protein standard. 

Laccase activity was routinely assayed spectrophotometrically, following the oxidation of syringaldazine substrate to tetramethoxy-azobis-methylene-quinone (II) at 530 nm. Assays were conducted at 70 °C in a volume of 3 mL containing 0.1 M potassium phosphate buffer pH 6.0 and 0.216 mM syringaldazine. The reaction was initiated by the addition of FNTL enzyme, after preincubation of the reaction mixture at 70 °C for 2 min. The reaction was monitored by measuring the change in absorbance at 530 nm over time using a UV–visible spectrophotometer (Shimadzu). One unit (U) of laccase activity was defined as a change in absorbance at 530 nm of 0.001 per minute under the assay conditions [50,51]. 

### 2.6. Quantitative Evaluation of Antibiotic Biodegradation by FNTL 

The actual capability of the FNTL enzyme to act on structurally different antibiotics from tetracyclines: TC and OTC; β-lactams: AMX; and fluoroquinolone: CIP was evaluated using the natural redox mediator AS (all chemicals from Sigma-Aldrich, St. Louis, MO, USA).

TC was dissolved in distilled water, while OTC, AMX, and CIP were dissolved in 0.1 N HCl. Biodegradation reactions were conducted in a volume of 500 µL containing a universal buffer UB4 (20 mM HEPES, 20 mM MES, and 20 mM sodium acetate) [65] at pH 6.0. Each reaction contains the partially purified FNTL (specific activity of 454,902 U mg^−1^ at 70 °C and pH 6.0, with syringaldazine) to a final protein concentration of 0.1 mg mL^−1^, the corresponding antibiotic at 0.5 mg mL^−1^, and 0.5 mM AS redox mediator. The reaction tubes were tightly closed and incubated with shaking at 180 rpm at 40, 30, and 20 °C for 24 h.

The assays were conducted in triplicate, and controls were treated equally without the addition of enzyme or mediator. After incubation, acetonitrile was added in a 3:1 ratio to stop the reactions. These were then centrifuged at 17,310× *g* for 20 min at 4 °C, and the supernatants were stored at −20 °C prior to analysis by high-efficiency liquid chromatography with a diode array detector (HPLC-DAD). 

The biodegradation of antibiotics was determined using a Shimadzu LC-20AT HPLC-DAD system (Shimadzu, Corporation, Kyoto, Japan). The separation of the analytes was carried out on a reverse-phase NUCLEODUR 100-5 C18 ec column (Macherey-Nagel, Düren, Germany, with a particle size of 5.0 µm, a length of 250 mm, and an internal diameter of 4.6 mm), with elution under isocratic conditions at 45 °C. A mixture of 75% 0.02 M oxalic acid, 18% acetonitrile, and 7% methanol was used at a flow rate of 1 mL min^−1^. The mobile phase, acidified with oxalic acid, was used to prevent the appearance of tailing peaks [66,67].

Biodegradation reaction samples were thawed, filtered through a 0.22 mm filter (Merck Millipore, Burlington, MA, USA), placed in amber HPLC vials, and injected (25 µL). Chromatograms were obtained using a diode array detector model SPD-M20A, with each antibiotic being analyzed at different wavelengths: 254 nm for TC and OTC, 235 nm for AMX, and 273 nm for CIP. The specific retention times for each of the control antibiotics with no enzymatic treatment were: 5.2, 4.4, 2.7 and 4.5 min for TC, OTC, AMX and CIP respectively. The analysis of elution peak areas was performed using Shimadzu’s LabSolutions/LCsolution software 1.23 platform. The percentage of antibiotic biodegradation was calculated by comparing the elution peak areas of the sample chromatograms with those of the controls containing only antibiotics, incubated under the same experimental conditions as the samples treated with the enzyme and mediators (Figure S2). The following equation was used: (% Antibiotic biodegradation = [(control peak area − sample peak area)/control peak area] × 100) [68].

### 2.7. Preliminary Evaluation of the Antimicrobial Effect of Antibiotics Treated with FNTL

The antimicrobial effect of selected antibiotics (TC, OTC, AMX, AMP, and CIP) was evaluated using AS as natural redox mediator. The ecotoxicity of their biotransformation products after enzymatic treatment with FNTL was evaluated with sensitive test bacteria: *E. coli* C41(DE3), routinely cultured in LB medium at 30 °C, and *B. subtilis*, cultured in HVMOD-D1 medium pH 6.5 (NaCl 48 g L^−1^, glucose 5 g L^−1^, yeast extract 4 g L^−1^, peptone 4 g L^−1^, casamino acids 3.5 g L^−1^, sodium citrate 2 g L^−1^, MgSO_4_ × 7H_2_O 4 g L^−1^, CaCl_2_ 0.05 g L^−1^, K_2_HPO_4_ 0.4 g L^−1^, Fe(NH_4_)_2_(SO_4_)_2_ × 6H_2_O 0.04 g L^−1^) at 30 °C. 

Biodegradation reactions were conducted as described in Section 2.6, except for the concentration of CIP, which was 0.0001 mg mL^−1^ for the assays with *E. coli* C41(DE3), due to its high sensitivity to this antibiotic. 

For visualization of the results, a 100 µL bacterial lawn (prepared according to the McFarland 0.5 standard, which corresponds to OD_600_ = 0.1 [69]) was spread on the respective solid media. Then, sterilized 6 mm filter paper disks with 5 µL of each biodegradation reaction or controls without the addition of enzyme or mediator were placed on the inoculated agar plates, which were incubated at 30 °C for 18 h. 

### 2.8. Statistical Analysis

The statistical analysis was performed using GraphPad Prism software (Version 8.0.2, GraphPad Software, Boston, MA, USA, 2019). The results were expressed as the mean of triplicates ± standard deviation (SD). The statistical significance was determined by evaluating the *p* value with a 95% significance level obtained using a two-way analysis of variance (ANOVA), followed by multiple comparisons between the treatments and control group using the Dunnett post-hoc test.

## 3. Results

### 3.1. In Silico Evaluation of Antibiotic Affinity to the Spore-Coat Laccase of Bacillus sp. FNT (FNTL)

#### 3.1.1. Homology Modeling of FNTL

Due to the fact that the crystallographic structure of the spore-coat laccase from *Bacillus* sp. FNT has not been solved yet, a three-dimensional model was generated using the modeling method implemented in the SWISS-MODEL server, using as a template the AlphaFold v2 model of the multicopper oxidase A0A6I7FGV2 from *Bacillus* sp. NSP91, which shares 84% identity with the *fntlac* gene sequence with 100% coverage. The resulting model exhibited a Global Mean Quality Estimation (GMQE) score of 0.97. Further assessments of 3D stereochemical quality performed to verify the reliability of the model for docking studies revealed that 97% of the residues were in Ramachandran-favored regions (Figure S3a). When compared with a non-redundant set of PDB structures, the resulting model QMEAN4 score fell within the range of |Z-score| < 1, aligning with the distribution observed for most of the high-resolution protein crystal structures (Figure S3c). The high quality of the three-dimensional model generated was further confirmed by MolProbity and QMEANDisCo quality scores (Table S1). These results validate both the quality and stereochemistry of the model, which indicates that it is suitable for conducting docking experiments. A second model built with SWISS-MODEL using the 1GSK crystal of CotA laccase (*B. subtilis*) as template was discarded due to its inferior quality (Table S1). 

**Figure 1:**
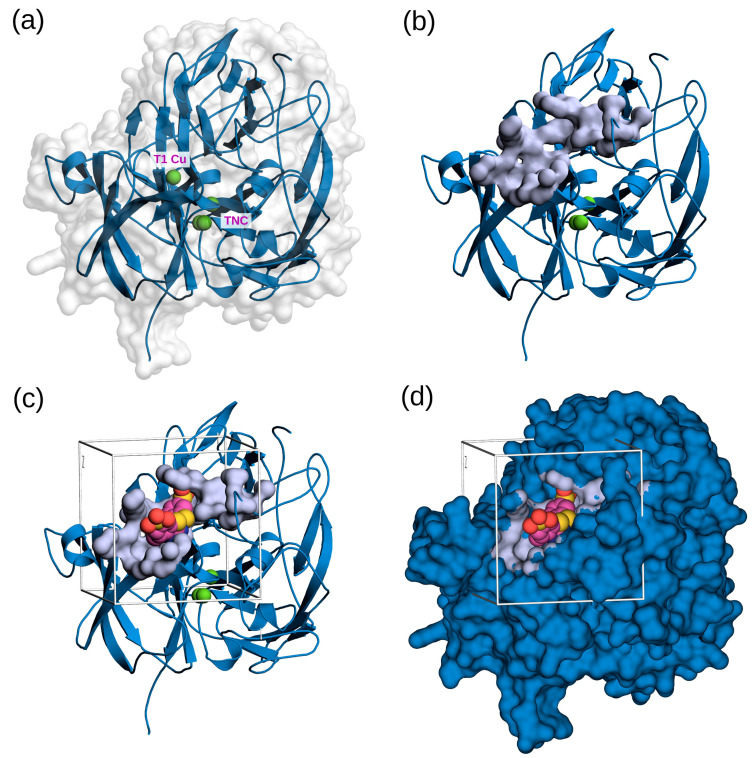
Three-dimensional representation of the structural model of FNTL and the ligand-binding pocket used for docking experiments. (**a**) Smoothed cartoon-style representation of the 3D model of FNTL. The four copper atoms in the active center of the FNTL are shown in green, with the top atom representing the mononuclear copper center (T1 Cu) and the bottom three representing the trinuclear copper site (TNC). The surface of the enzyme is depicted in white. (**b**) Surface representation of the residues, forming the ligand-binding pocket subcavity at the T1 Cu site, as identified by CASTp. (**c**,**d**) Theoretical positioning of ABTS (in van der Waals representation) within the ligand-binding pocket at the T1 Cu site. The boxes in (**c**,**d**) illustrate the selected sampling site for docking runs. The coordinates of the copper atoms and ABTS were inferred from the crystal structure of *B. subtilis* cotA laccase (PDB ID: 1OF0), after structural alignment. All the molecular representations were generated using PyMOL.

According to the modeled structure of FNTL (Figure 1), the overall fold is very similar to that of other bacterial laccases determined by X-ray crystallography (e.g., CotA from *B. subtilis* [70] and CueO from *E. coli* [71]). It contains three cupredoxin-like domains (each composed of a β-sandwich with seven strands in 2 β-sheets, structured in a Greek key beta-barrel), and the four copper atoms in the catalytic center, which are characteristic of these enzymes. Based on their unique spectroscopic features, they are divided into three types of structurally and functionally distinct copper sites: Type 1 (T1), Type 2 (T2), and binuclear Type 3 (T3), arranged as a mononuclear copper center (T1), and one trinuclear copper center TNC (T2/T3) [72] (Figure 1a). T1 is located near the substrate-binding site and accepts electrons from the reducing substrate, while the TNC binds oxygen and reduces it to water [73].

#### 3.1.2. Molecular Docking Experiments

Molecular docking simulations were performed to explore the interaction and affinities of the nine different antibiotics with the putative substrate-binding pocket of FNTL located near the mononuclear T1 Cu site, where substrate oxidation occurs in laccases [72,74]. According to CASTp, this binding pocket is composed of three subpockets, which together were predicted to have approximately 313.696 Å^2^ of solvent-accessible surface area and a volume of 269.531 Å^3^ (Figure 1b). The theoretical placement of ABTS (a model substrate often co-crystallized with other laccases) within the ligand-binding pocket at the T1 Cu site was confirmed by structure superimposition. The coordinates of this ligand were used as a reference, in conjunction with the CASTp predicted pockets, to set the grid for conducting the docking calculations, as shown in Figure 1c,d.

**Figure 2:**
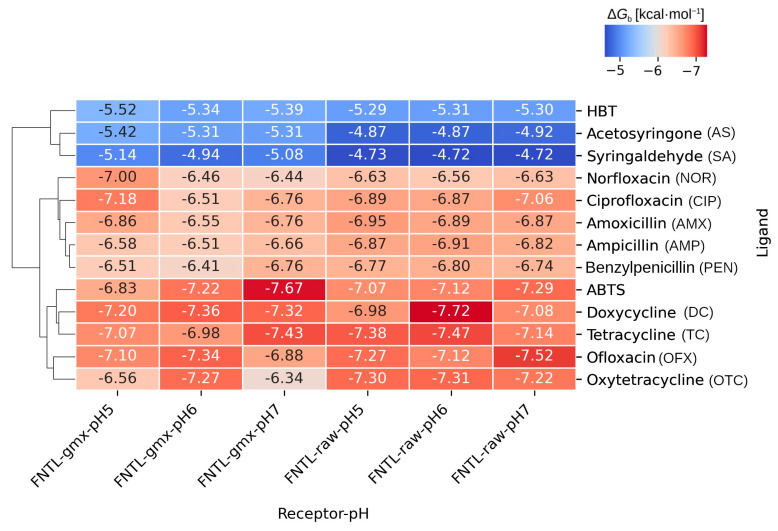
Heatmap showing the average free energies of binding, Δ*G_b_* (kcal mol^−1^), obtained for each ligand, receptor, and pH combination in the docking experiments. Each cell represents the average energy obtained from 10 independent technical replicates. ’FNTL-gmx’ corresponds to the Gromacs energy-minimized model, and ‘FNTL-raw’ corresponds to the non-minimized model of FNTL. The dendrogram on the left represents the hierarchical clustering performed with the Seaborn Clustermap function using the average Euclidean distance.

The molecular docking analyses were carried out at three different pH values (5.0, 6.0, and 7.0). Thus, 780 docking calculations were performed, considering 10 technical replicates for each combination of receptor, ligand, and pH. The estimated average free binding energies resulting for each system are condensed in the heatmap of Figure 2.

When examining the binding energy results (Figure 2), it becomes apparent that the gromacs-minimized receptor (FNTL-gmx) generally exhibited more favorable binding energies with various ligands compared to the non-minimized model (FNTL-raw). This is evidenced by consistently lower average binding energy values, which indicates that FNTL-gmx is more conducive to stronger ligand interactions. Consequently, all subsequent analyses are based on energy results derived from this latter model.

As expected, the different chemical structures of the studied ligands resulted in distinct affinity patterns towards the binding site of FNTL, as revealed by their free energies of binding (Δ*G*_b_). The clustering of these energies in the heatmap of Figure 2 clearly shows a distinct variation among different substrates, categorized into three main clusters. The first cluster is characterized by the weakest affinity, comprising the redox mediators HBT, AS, and SA. The second cluster, with mid-range affinity, encompasses all β-lactam antibiotics along with the fluoroquinolones NOR and CIP. Lastly, the third cluster with the highest affinity ligands is formed by all the tetracyclines, the fluoroquinolone OFX, and the synthetic redox mediator ABTS. 

When focusing solely on the binding energy results of the different antibiotics, it is evident that DC consistently exhibited the lowest average energy across all pH levels, suggesting a strong and stable binding affinity to FNTL. On the other hand, PEN consistently showed relatively higher average energy values, indicating comparatively weaker binding. 

The analysis also revealed significant variations in the affinity of the different ligands across the three pH conditions evaluated. Some antibiotics showed notable variations in their average binding energy as a function of pH, such as OTC, CIP, and NOR, suggesting that the binding affinity of these antibiotics to the enzyme is pH-dependent. 

In contrast, antibiotics from the β-lactams group showed more consistent energy values across the three different pH values, presenting very little variation and suggesting less pronounced pH sensitivity. The strongest affinity was predicted with AMX at pH 5.0 (−6.86 kcal mol^−1^). 

A closer look at the tetracycline group evidenced that the strongest affinity was reached with TC at pH 7.0 (−7.43 kcal mol^−1^), followed by DC and OTC at the same pH. However, this preference is modified at lower pH values, with DC showing the highest affinity of the tetracyclines group at pH 6.0 and 5.0 (−7.36 and −7.20 kcal mol^−1^, respectively).

For the fluoroquinolones, while OFX was clustered together with the highest affinity ligands, having the strongest affinity at pH 6.0 (−7.34 kcal mol^−1^), CIP and NOR were clustered together with β-lactam antibiotics in the second cluster, with their strongest affinity at pH 5.0 (−7.18 and −7.00 kcal mol^−1^, respectively). 

Regarding the different redox mediators, ABTS at pH 7.0 exhibits the highest affinity of all ligands (−7.67 kcal mol^−1^), while HBT, AS, and SA, grouped together in the first cluster, reached their maximum affinities at pH 5.0 (−5.52, −5.42, and −5.14 kcal mol^−1^, respectively), with SA exhibiting the weakest affinity of all the different ligands evaluated (−4.94 kcal mol^−1^ at pH 6.0).

**Figure 3:**
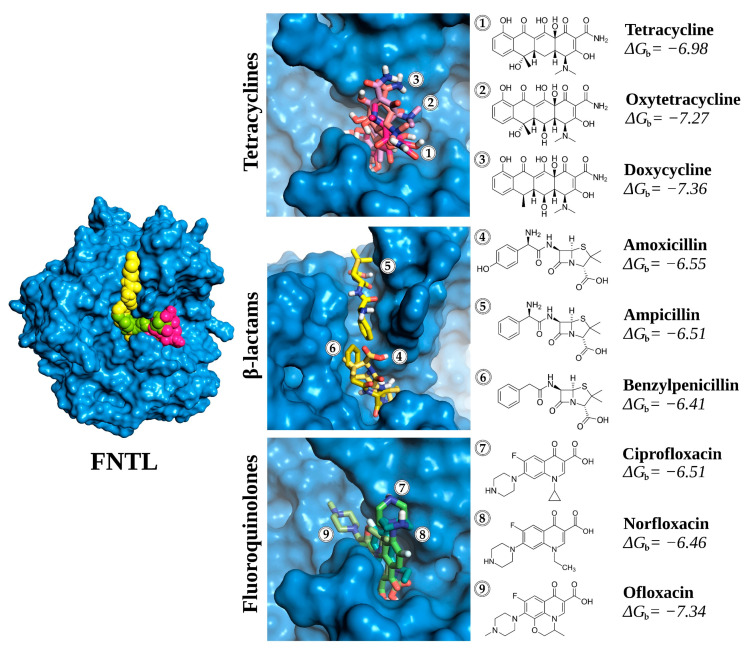
Three-dimensional representation of the conformations of the antibiotic-FNTL complexes resulting from docking at pH 6.0. Tetracycline group (in magentas): (1) tetracycline (TC), (2) oxytetracycline (OTC), and (3) doxycycline (DC); β-lactams group (in yellows): (4) amoxicillin (AMX), (5) ampicillin (AMP), and (6) benzylpenicillin (PEN); and fluoroquinolone group (in greens): (7) ciprofloxacin (CIP), (8) norfloxacin (NOR), and (9) ofloxacin (OFX). The values of free binding energy (Δ*G*_b_) are expressed in kcal mol^−1^.

Considering these results, there does not appear to be a clear or consistent pH-dependent trend, so it was decided to continue with the analysis of the molecular docking results obtained only at pH 6.0. The modeled enzyme active site and predicted binding of antibiotics from the three different families are shown in Figure 3.

#### 3.1.3. Analysis of Receptor–Ligand Interactions

**Figure 4:**
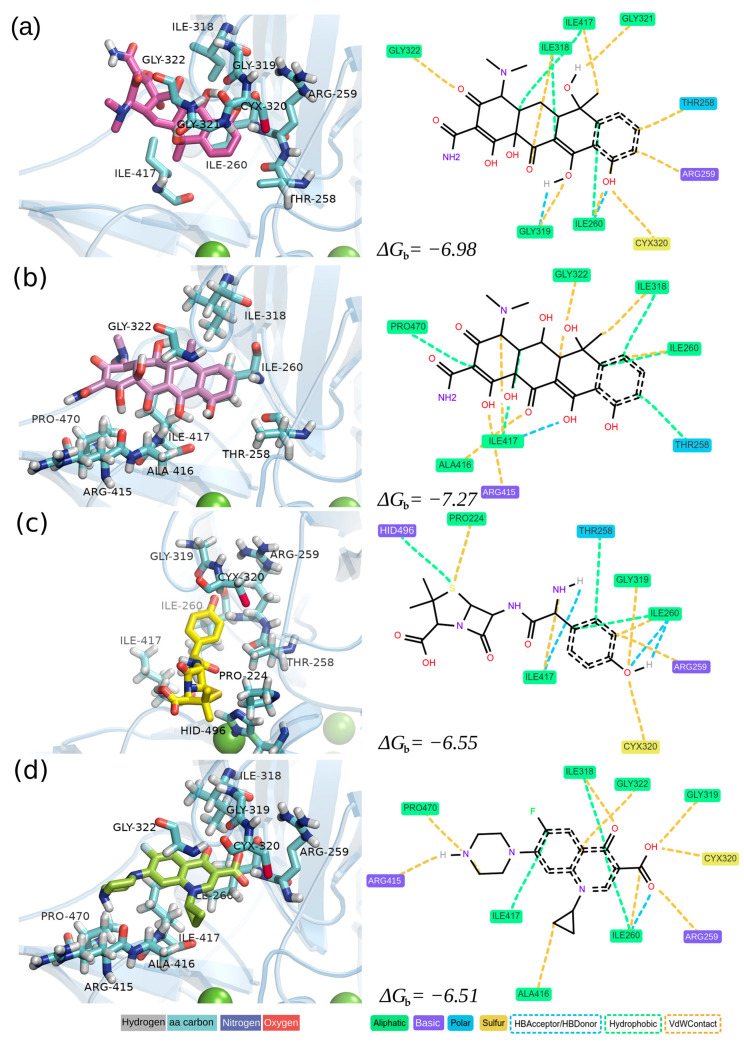
Different representations of the antibiotic-FNTL complexes showing the interactions identified between the three-dimensional model of the laccase enzyme and the antibiotics (**a**) TC, (**b**) OTC, (**c**) AMX, and (**d**) CIP resulting from docking experiments. Molecular interaction maps were constructed with ProLIF using the “aggregate” function to discard low-frequency interactions and a 6 Å cutoff to infer interactions. The estimated free energies of binding (kcal mol^−1^) are shown at the bottom of each plot.

To delve deeper into the protein–ligand interactions resulting from molecular docking experiments, representatives from each antibiotic family, namely TC, OTC, AMX, and CIP, were chosen based on their high global consumption [7,75,76]. The binding affinity between a ligand and the active site of an enzyme depends on various factors, including hydrophobic and electrostatic interactions, hydrogen bonding, and van der Waals forces. For instance, hydrogen bonding involving basic and polar residues is crucial for stabilizing the binding interaction and significantly contributes to the overall binding affinity. Therefore, to identify the primary interaction types between the binding site of FNTL and the docked antibiotics, we conducted an interaction analysis at pH 6.0 using ProLIF (Figure 4). 

The detailed analysis of molecular interactions unveiled distinct patterns for ligand binding (refer to Figure 4 and Table 2 for details). Hydrophobic interactions were observed with residues PRO224, THR258, ILE260, ILE318, ILE417, PRO470, and HIS496, indicating their pivotal role in stabilizing ligands within the active site. Notably, persistent hydrophobic contacts with ILE260, interacting with all four antibiotics, suggest its crucial role in creating a hydrophobic pocket conducive to binding diverse antibiotics. Van der Waals (VdW) contacts were prominent, especially with residues PRO224, THR258, ARG259, ILE260, ILE318, GLY319, CYS320, GLY322, ARG415, ALA416, ILE417, PRO470, and HID496. ARG259 and ILE260 exhibited substantial VdW contacts across all antibiotics, emphasizing their significance in determining the ligand–enzyme complex stability. The correlation between the abundance of VdW interactions and binding energies reinforces the notion that these non-covalent forces play a crucial role in determining overall binding affinity. Hydrogen bonding interactions were observed with THR258, ILE260, GLY319, and ILE417, with ILE260 specifically engaging in hydrogen bonding only with AMX. 

The difference between the antibiotics with weaker affinity (CIP and AMX) and those with stronger affinity (TC and OTC) can be attributed to the nature and extent of molecular interactions observed in the active site of FNTL. In the case of CIP and AMX, despite favorable interactions, such as van der Waals contacts and hydrogen bonding, the magnitude or quantity of these interactions might not be substantial enough to significantly contribute to more favorable binding energies. Conversely, TC and OTC exhibit more negative energies of binding, indicating a stronger interaction. Hydrophobic interactions, especially in residues like ILE260, ILE318, and ILE417, consistently present in both antibiotics, could contribute significantly. Additionally, the formation of hydrogen bonds and van der Waals contacts in these residues may also be more pronounced, contributing to the observed higher affinity. It is important to emphasize that a synergistic effect generated by different types of interactions, such as hydrophobic and hydrogen bonds, might contribute to a more negative binding energy. In this context, specific interactions with key residues, such as ILE260, could significantly influence the variation in affinity between the different antibiotics analyzed.

### 3.2. Experimental Evaluation of Antibiotic Biodegradation by FNTL

In order to evaluate the actual capability of FNTL to utilize structurally different antibiotics as substrates, the same representatives of the tetracyclines, β-lactams, and fluoroquinolones families selected for receptor–ligand interaction analysis (TC, OTC, AMX, and CIP) were used to perform biodegradation and ecotoxicity assays at different temperatures: 40, 30, and 20 °C at pH 6.0 for 24 h with and without AS.

For this, the laccase-encoding gene (*fntlac*) from *Bacillus* sp. FNT was first overexpressed in a soluble, catalytically active form in *E. coli* BL21, and the cell-free extract was heat denatured (at 85 °C for 5 min) to obtain the semi-purified FNTL (Figure S1). The recombinant enzyme exhibits remarkably high specific activity (454,902 U mg^−1^) at 70 °C and pH 6.0, using syringaldazine substrate, and according to our previous study, it is expected to possess >75,000 U mg^−1^ at 20 °C and pH 6.0 [50]. 

**Figure 5:**
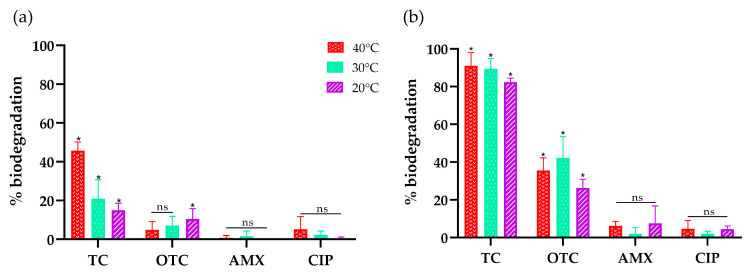
Percentage of biodegradation after 24 h reaction in the presence and absence of the natural redox mediator AS. Assays were performed with four highly consumed antibiotics representative of the different families studied (TC, OTC, AMX, and CIP) at three temperatures: 40 °C (red), 30 °C (light green), and 20 °C (purple). (**a**) Direct assays performed with FNTL (no redox mediator). (**b**) Indirect assays performed with FNTL and AS. Each assay was carried out in triplicate, and statistical analysis was performed with ANOVA and Dunnett´s post-test, where (*) *p* < 0.05 corresponds to a significant value and (ns) a non-significant value with respect to the control.

Results shown in Figure 5 indicate that, for TC, incubation with the natural redox mediator AS results in 91, 90, and 82% of biodegradation at 40, 30, and 20 °C, respectively, whereas direct oxidation reached 46, 21, and 15% at the same temperatures. In the case of OTC, it was also utilized by FNTL, although to a lesser extent, reaching 36, 42, and 26% of biodegradation at 40, 30, and 20 °C, respectively, whereas direct oxidation reached a maximum of 11% at 20 °C. Unfortunately, for both β-lactams and fluoroquinolone representatives, biodegradation was not statistically significant at any of the conditions tested.

It is worth noting that the molecular docking experiments were performed at standard conditions of temperature (25 °C) with no combination of antibiotics and redox mediators in the enzyme active site. Therefore, the average energies of binding obtained at pH 6.0 are comparable only with the results of direct oxidation assays at lower temperatures (Figure 5a), which seems to align well, as the stronger affinities were predicted for the tetracyclines and weaker affinities were predicted for AMX and CIP. Interestingly, experimental results with TC reached higher biodegradation rates than OTC at all the conditions tested, while according to the molecular docking results at pH 6.0, the average energies of binding predicted a stronger affinity of FNTL with OTC than TC at that pH.

In addition, the antimicrobial effect of antibiotics and the ecotoxicity of their biotransformation products after enzymatic treatment with FNTL were qualitatively assessed on sensitive *E. coli* and *B. subtilis* strains. The absence or reduction in the growth inhibition zone caused by the different antibiotics is an indication of a loss of antimicrobial efficacy after enzymatic treatment [8] and suggests that the biotransformation products are not toxic to the bacterial cells. On the contrary, an increase could indicate that biotransformation of antibiotics by FNTL action could be yielding toxic products that also affect bacterial growth.

**Figure 6:**
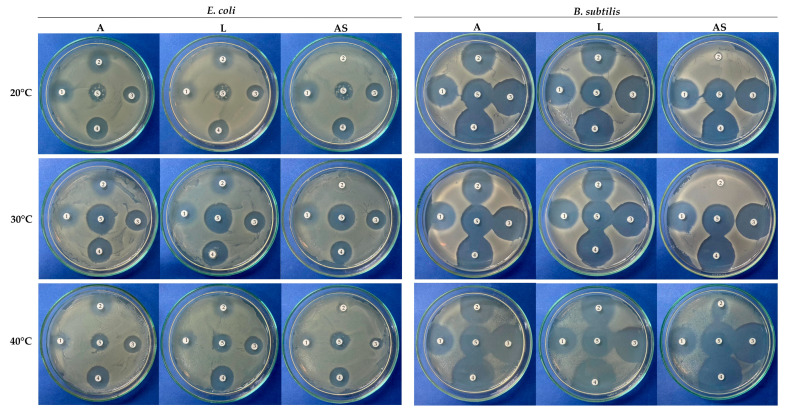
Qualitative evaluation of enzymatic treatment of different antibiotics with FNTL and its ecotoxicity effect in *E. coli* and *B. subtilis* at 20, 30, and 40 °C at pH 6.0 for 24 h. (**A**): control for each antibiotic: (1) OTC, (2) TC, (3) AMX, (4) AMP, and (5) CIP. All at 0.5 mg mL^−1^ except CIP with *E. coli* at 0.0001 mg mL^−1^; (**L**): direct biodegradation assays with FNTL (0.1 mg mL^−1^) with no redox mediator; (**AS**): indirect assays with FNTL plus 0.5 mM AS redox mediator. For visualization of the results, a 100 µL bacterial lawn of the test bacteria was spread on LB agar media, and sterilized filter paper disks with 5 µL of each biodegradation reaction were placed on the inoculated agar plates and incubated at 30 °C for 18 h. Each assay was performed in triplicate.

The results shown in Figure 6 indicate a complete reduction in the TC growth inhibition halo after 24 h of incubation with FNTL-AS at 40, 30, and 20 °C and pH 6.0, for both *E. coli* and *B. subtilis*, while direct oxidation did not cause the antimicrobial effect of this antibiotic to be lost. Interestingly, no changes in the size of the halo were observed for OTC at any of the conditions tested, which will require further analysis to clarify whether the biotransformation products might be toxic for the test bacteria.

In the case of AMX and CIP, no significant changes were observed in their growth inhibition zones, in agreement with the results obtained by molecular docking, enzyme active site interactions, and quantitative HPLC-DAD analysis, rather than suggesting ecotoxicity of the biotransformation products.

## 4. Discussion

The great versatility of laccases allows their use in very diverse areas, including the highly relevant ecological degradation of emerging and persistent environmental pollutants. Following the functional approach, which is based on the screening of enzymatic activities of interest from culturable microorganisms [77,78], it was possible to obtain a novel native spore-coat laccase from the thermoalkaliphilic bacterium *Bacillus* sp. FNT, isolated from an environmental sample collected from a hot spring in a geothermal site. Due to the low growth yield of extremophiles and the complexity of protein extraction from spores, the recombinant version was developed, and the enzyme has been previously characterized and evaluated against several structurally different substrates (i.e., syringaldazine, gallic acid, ABTS, Congo red, methyl orange, methyl red, Coomassie brilliant blue R250, bromophenol blue, malachite green, crystal violet, Remazol brilliant blue R, anthracene, benzo[a]anthracene, and benzo[a]pyrene), highlighting its high activity and versatility [50,51].

It has been previously reported that at 80 °C, this thermophilic enzyme reaches its optimum activity with syringaldazine, being also highly active in a wide range of temperatures (20–90 °C), maintaining over 50% of catalytic activity at temperatures between 50 °C and 90 °C and less than 40% of activity at temperatures below 40 °C. Nevertheless, due to its remarkably catalytic activity, FNTL still exhibits very high specific activity at 20 °C (at only 15% of its optimum at 80 °C) [50]. Thus, aiming to evaluate the biotechnological potential of this extremozyme for wastewater bioremediation, taking into account operability and economy, previous biotransformation experiments of recalcitrant environmental pollutants have been performed at 40 °C. As a result, FNTL was able to biodecolorize eight synthetic dyes (50 mg L^−1^), in 30 min at pH 6.0 in the presence of AS [50] and oxidize over 40% of anthracene (0.05 mg mL^−1^) after 8 days at pH 5.0 with the aid of ABTS [51]. 

Then, in order to better understand how these interesting properties relate to the enzyme’s three-dimensional structure, crystallization trials were performed with FNTL (purified to homogeneity and highly concentrated up to 100 mg mL^−1^). The enzyme was screened for protein crystallization conditions with commercial kits (Molecular Dimensions Ltd., Sheffield, UK; Hampton Research Corp., Aliso Viejo, CA, USA) using an ARI Gryphon crystallization robot (Art Robbins Instruments LLC) by the sitting-drop vapor-diffusion method. After several unsuccessful attempts to obtain good-quality crystals, to date, no X-ray crystal structure has been determined for this interesting extremozyme. 

Therefore, in this study, we constructed a high-quality three-dimensional model of FNTL using SWISS-MODEL and the multicopper oxidase A0A6I7FGV2 from *Bacillus* sp. NSP91 as a template. Subsequently, we sought to establish a correlation between the potential of FNTL for antibiotic biodegradation and binding energies derived from molecular docking experiments for various commonly used antibiotic families, including tetracyclines, β-lactams, and fluoroquinolones. As the use of redox mediators in conjunction with laccases expands the range of substrates susceptible to enzymatic oxidation through indirect catalysis, thereby facilitating the degradation of recalcitrant compounds [44], the binding of four different redox mediators (AS, SA, HBT, and ABTS) to FNTL was also evaluated.

The results revealed significant variations in the free energies of binding of different substrates within the FNTL binding pocket, which were categorized into three clusters: one with the lowest affinity (HBT, AS, and SA), another with intermediate affinity (AMX, AMP, PEN, NOR, and CIP), and the third with the highest affinity ligands (TC, OTC, DC, OFX, and ABTS). DC consistently exhibited the lowest average energy, indicating a robust and stable binding affinity to FNTL, while PEN exhibited relatively higher average energy values, indicating weaker binding. Interestingly, DC and AMP antibiotics showed minimal variation in predicted binding energies across three different pH conditions, while OTC, CIP, and NOR showed the largest pH-dependent variations in binding energy (albeit within 15% of their optimal result), indicating sensitivity to pH changes. Although no clear or consistent pH-dependent trend was observed among the different ligands, the variability in pH dependence suggests a complex interplay of electrostatic, hydrogen bonding, and hydrophobic interactions within the binding pocket, highlighting the importance of protonation states in substrate–enzyme interactions. 

To further explore protein–ligand interactions with highly consumed antibiotics, representatives of each family (TC, OTC, AMX, and CIP) were selected for experimental analysis with FNTL at pH 6.0 and different temperatures (40, 30, and 20 °C), with and without the addition of AS. 

Even though the predicted affinity was stronger with the most widely used artificial redox mediators, ABTS and HBT, their use was discarded because of their economic infeasibility and long-term enzyme toxicity [76]. On the other hand, AS natural origin, derived from lignin, a renewable source, allows for more environmentally friendly and very economical processes. Upon oxidation, AS is expected to form a phenoxy radical that acts as a radical species, abstracting a proton and an electron from the target substrate [79]. The stability and radical formation efficiency of AS, attributed to the methoxy substituents (-O-CH_3_) in its phenolic structure, make it an optimal natural mediator for laccase studies [80]. Of note is the exclusion of SA as a redox mediator, not only because of its weakest affinity (−4.94 kcal mol^−1^ at pH 6.0) but also because previous studies have reported potentially higher toxicity of the degradation products resulting from the biotransformation of antibiotics with laccase and SA. This toxicity could result from the oxidation of aromatic structures, especially phenols, to quinonoids [81].

The HPLC results confirmed the high versatility of this extremozyme and preference towards antibiotics from the tetracycline family, as predicted by molecular docking analysis. For tetracyclines, FNTL with AS results in 91, 90, and 82% of biotransformation of TC at 40, 30, and 20 °C and pH 6.0, respectively, whereas it reached 46, 21, and 15% at the same conditions with no mediator aid. In the case of OTC, biotransformation reached 36, 42, and 26% at 40, 30, and 20 °C and pH 6.0, respectively, whereas the enzyme alone reached a maximum of 11% at 20 °C, indicating that FNTL requires the addition of a redox mediator for the removal of OTC. These results are in agreement with previous studies indicating that significant antibiotic elimination by laccases usually requires the involvement of an appropriate mediator [40,82].

Tetracyclines ranked second worldwide in production and usage, while first in China [76,83]. They are broad-spectrum antibiotics derived from various *Streptomyces* species that can inhibit bacterial protein synthesis by preventing the binding of aminoacyl-tRNA with bacterial ribosomes [84]. Members of this family are characterized by a chemical structure consisting of a naphthacene core comprising four linearly fused six-membered carbocyclic rings with various functional groups in their structure, which are responsible for their distinctive properties [84]. For instance, the presence of hydroxyl (-OH) groups generates similarity with phenolic structures, which are part of the natural substrates of laccases. 

Based on receptor–ligand interaction analysis, TC binds near the T1 Cu site, interacting with aliphatic residues, including glycine, leucine, alanine, and isoleucine, which dominate the interactions, fostering hydrophobic contacts and van der Waals forces in direct oxidation assays. Notably, OTC, which contains a hydroxyl (-OH) group in its structure that is not present in TC, exhibits a unique set of nearby polar residues (serine, glutamine, and asparagine), suggesting a more varied interaction profile compared to the other antibiotics. These differences may have implications for catalysis, as it was experimentally observed that TC oxidizes more effectively than OTC at pH 6.0, despite OTC having a stronger predicted affinity than TC at that pH (−7.27 and –6.98 kcal mol^−1^, respectively). 

Remarkably, this research study indicates that FNTL exhibited a higher antibiotic biodegradation capability when compared to other laccases documented in the literature, highlighting its great biotechnological potential for application in wastewater bioremediation. Especially when comparing the antibiotic concentrations tested (0.5 mg mL^−1^), FNTL is one of the laccases with the highest tetracyclines biodegradation capacity reported, at a concentration much higher than that generally found in the environment. For example, TC has been found to be present at concentrations ranging from 50 to 850 ng L^−1^ in 80% of the effluents of seven sewage treatment plants in Wisconsin, US [85].

To date, fungal laccases are more frequently used than bacterial laccases in antibiotic biodegradation studies; for instance, the laccase from *Trametes versicolor* (IFO−6482) with no mediator was able to biodegrade approximately 16% and 14% of TC and OTC (0.04 mg mL^−1^), respectively, after 4 h at 30 °C and pH 4.5 with shaking at 150 rpm [86]. Also, experiments with the commercial laccase from *T. versicolor* (Sigma-Aldrich 51639), in the absence of redox mediators, reached 78% removal of TC (0.1 mg mL^−1^) after 18 h at 30 °C [87]. Another example is the native laccase (Lac-Q) from the white-rot fungus *Pycnoporus* sp. SYBC-L10 used for biodegradation assays of TC and OTC (0.05 mg mL^−1^) with 0.5 mM of different redox mediators; Lac-Q–ABTS system showed the highest degradation rates, with a removal ratio of 96% of TC and a removal ratio of 90% of OTC after only 3 min of static incubation at 25 °C and pH 6.0, while the Lac-Q–SA system had the second highest removal treatment, with a removal ratio of 49% of TC and 45% of OTC at the same conditions; however, no reduction in TC and OTC was obtained after treatment with Lac-Q without any mediator [88]. On the other hand, the recombinant bacterial laccase from *Bacillus amyloliquefaciens* was able to biodegrade 37.5% of TC (0.1 mg mL^−1^) after 12 h at 30 °C and pH 7.0 [89], while using the recombinant laccase from *B. subtilis* TJ-102, it was possible to almost completely biodegrade TC (0.1 mg mL^−1^) after 2 h at 25 °C [8].

In addition, it has been reported that antibiotic degradation by laccases mostly leads to reduced toxicity [40]. For example, the treatment with fungal laccase from *T. versicolor* and HBT successfully removed the growth inhibition of TC towards test bacteria: *E. coli* and *B. subtilis,* as well as the algae *P. subcapitata*, after 1 h at 30 °C and pH 4.5 [86]. Also, Lac-Q from *Pycnoporus* sp. SYBC-L10 with ABTS showed that the antimicrobial activity of TC and OTC and their transformation products on *E. coli* and *Bacillus altitudinis* was completely removed after 5 min of treatment at 25 °C [88]. However, in other cases, the toxicity observed in test bacteria may also be related to the incomplete degradation of the antibiotic [8].

In this study, the antimicrobial effect of each antibiotic and the ecotoxicity of their biotransformation products after the enzymatic treatment with FNTL were qualitatively assessed on Gram-negative *E. coli* and Gram-positive *B. subtilis*. According to the results, there was a complete reduction in the growth inhibition halo, indicating the loss of the antimicrobial effect, and no ecotoxicity was observed for TC after 24 h of incubation with AS at 20 and 30 °C and pH 6.0 for both bacterial strains and at 40 °C with *E. coli* (Figure 6). Interestingly, with *B. subtilis,* it was possible to observe a halo surrounding TC treated with FNTL-AS at 40 °C, which, although smaller than the antimicrobial activity of the untreated antibiotic control, might indicate that the biotransformation products generated at higher temperatures under the assay conditions could be toxic for Gram-positive bacteria. Also, further analysis will be necessary to elucidate whether the biotransformation products of OTC are actually toxic for the test bacteria or could be due to the incomplete biodegradation of this antibiotic, since no changes in the halo size were observed at any of the different experimental conditions. As mentioned above, it should be noted that the antibiotic concentrations used for the biodegradation tests (0.5 mg mL^−1^) were higher than those commonly cited in the literature and those found in the environment. However, due to the high sensitivity of *E. coli* C41(DE3) to CIP, the final concentration selected for this antibiotic in the qualitative assay was 0.0001 mg mL^−1^, and colony growth within the zone of growth inhibition was observed, which was reproducible and attributable to the low concentration of antibiotic used rather than contamination.

Future studies need to be directed towards establishing the nature and toxicity levels of the enzyme-generated degradation products. It has been proposed that a putative mechanism of TC biotransformation by laccases involves the opening of the aromatic ring to form small acidic molecules, which results in a loss of antibiotic activity [82]. The main transformation products of TC biodegradation by the action of the bacterial laccase from *B. amyloliquefaciens* have been identified by LC-MS, and the authors proposed three putative pathways for TC degradation [89]. In the first, the methyl group (C6) of TC was removed, and successive hydroxylation reactions occurred on C5 and C11a to generate TC1. It is worth noting that oxidation at the C5 position results in the formation of the corresponding ketone, OTC, and previous studies have shown that TC is first oxidized to OTC as the major transformation product [88,90,91]. In the second pathway, epimerization happened at position C4 to form 4-epi-TC (TC2), which was reported to be an important initial transformation process for tetracyclines [92]. Then TC2 could be decomposed to TC4 by demethylation, deamination, dehydrogenation, and hydroxylation reactions. For the third route, TC3 was generated from TC via demethylation and dehydrogenation, and then it was deaminated and hydroxylated to TC4 by oxidation at C4 [8]. Subsequently, the hydroxyl at C6 was removed to form TC5, which could be further decomposed to produce TC6, TC7, and TC8 [89]. Similar reactions leading to the formation of degradation products with a smaller molecular size have been reported for biodegradation of TC by fungal laccases, [48,82,87].

In the case of β-lactam and fluoroquinolone antibiotic biodegradation by FNTL, the HPLC results were very modest, which was consistent with their average binding energies at pH 6.0 predicted by molecular docking (−6.55 and −6.51 kcal mol^−1^ for AMX and CIP, respectively). Both antibiotics belong to the mid-range affinity energy cluster, and experimentally, they did not achieve statistically significant biodegradation compared to their controls. In the case of CIP, the presence of both aliphatic and polar residues, including arginine and cysteine, introduces an additional layer of complexity, as the involvement of a sulfur-containing residue highlights potential interactions beyond conventional hydrophobic and polar contacts. In addition, no significant changes in the growth inhibition zones were observed for AMX and CIP, when qualitatively assessing ecotoxicity in *E. coli* and *B. subtilis,* which agrees with the in silico findings, rather than suggesting ecotoxicity of the biotransformation products. These results suggest that while biodegradation of tetracycline antibiotics by FNTL is efficient, especially with AS aid, elimination of β-lactams and fluoroquinolones is possible, although not sufficiently effective for application to their bioremediation, at least under the conditions tested. 

According to other studies, different immobilization techniques used with laccases have been demonstrated to enhance their activity, stability, and biodegradation performance compared to the use of free enzymes, and some of the advantages of immobilized laccases are convenience of operation, low cost, and reusability [8,89,93]. Therefore, the great biotechnological potential of this extremozyme could be further improved with future immobilization studies, especially with a view to its application in wastewater bioremediation. 

## Figures and Tables

**Table 1:** List of abbreviations.

Type	Molecule	Abbreviation
Artificial redox mediator	1-hydroxybenzotriazole	HBT
	2,2′-azino-bis(3-ethylbenzothiazoline-6-sulfonic acid)	ABTS
Natural redox mediator	Acetosyringone	AS
	Syringaldehyde	SA
Tetracycline antibiotics	Tetracycline	TC
	Oxytetracycline	OTC
	Doxycycline	DC
β-lactams antibiotics	Amoxicillin	AMX
	Ampicillin	AMP
	Benzylpenicillin	PEN
Fluoroquinolone antibiotics	Ciprofloxacin	CIP
	Norfloxacin	NOR
	Ofloxacin	OFX

**Table 2:** Molecular interaction fingerprints associated with antibiotic-FNTL complexes. The table showcases established interaction types between different antibiotics and residues within the binding pocket of the FNTL enzyme, denoted as ‘True’ for presence and ‘False’ for absence. The fingerprints were generated using ProLIF. Abbreviations: VdW (van der Waals); HB (hydrogen bond).

		Antibiotics			
Residue	Interaction	TC	OTC	AMX	CIP
PRO224	Hydrophobic	False	False	True	False
PRO224	VdWContact	False	False	True	False
THR258	Hydrophobic	True	True	True	False
THR258	VdWContact	True	False	False	False
ARG259	VdWContact	True	False	True	True
ILE260	Hydrophobic	True	True	True	True
ILE260	HBAcceptor	True	False	True	True
ILE260	HBDonor	False	False	True	False
ILE260	VdWContact	True	True	True	True
ILE318	Hydrophobic	True	True	False	True
ILE318	VdWContact	True	True	False	True
GLY319	HBDonor	True	False	False	False
GLY319	VdWContact	True	False	True	True
CYS320	VdWContact	True	False	True	True
GLY321	VdWContact	True	False	False	False
GLY322	VdWContact	True	True	False	True
ARG415	HBDonor	False	False	False	True
ARG415	VdWContact	False	True	False	True
ALA416	VdWContact	False	True	False	True
ILE417	Hydrophobic	True	True	False	True
ILE417	HBAcceptor	False	True	True	False
ILE417	HBDonor	False	False	True	False
ILE417	VdWContact	True	True	True	False
PRO470	Hydrophobic	False	True	False	False
PRO470	VdWContact	False	False	False	True
HIS496	Hydrophobic	False	False	True	False

## Data Availability

The data presented in this study are available on request from the corresponding author. The data are not publicly available due to privacy reasons.

## Supplementary Materials

The following supporting information can be downloaded at: https://www.mdpi.com/article/10.3390/biom14030369/s1, Figure S1: Electrophoretic analysis of the heterologous overexpression and partial purification of FNTL; Figure S2: Chromatograms of tetracycline separation by reversed-phase HPLC. Figure S3: Quality assessment of the 3D model of FNTL inferred by homology with SWISS-MODEL. Table S1: Quality scores of FNTL three-dimensional models generated with SWISS-MODEL.
