# Supplementary material for: Evaluation of Antibiotic Biodegradation by a Versatile and Highly Active Recombinant Laccase from the Thermoalkaliphilic Bacterium Bacillus sp. FNT
Source: Biomolecules. 2024 Mar 19;14(3):369. doi: 10.3390/biom14030369 (PMC10968384; doi:10.3390/biom14030369)
Supplement: Supplementary file 1 [file biomolecules-14-00369-s001.zip › biomolecules-2904117-supplementary.pdf]

biomolecules

Supplementary Materials

Evaluation of Antibiotic Biodegradation by a Versatile and Highly Active Recombinant Laccase from the Thermoalkaliphilic Bacterium *Bacillus* sp. FNT

Jorge Sánchez-SanMartín[2], Sebastián L. Márquez[2], Giannina Espina[2*], Rodrigo Cortés-Antiquera[1], Junsong Sun[3] Jenny M. Blamey[1,2*]

[1] Facultad de Química y Biología, Universidad de Santiago de Chile, Alameda 3363, Santiago 9170022, Chile.

[2] Fundación Biociencia, José Domingo Cañas 2280, Santiago 7750132, Chile.

[3] Green Chemical Engineering Technology R&D Center, Shanghai Advanced Research Institute, Chinese Academy of Sciences, Haike Road 99, Shanghai 201210, China.

* Correspondence: jblamey@bioscience.cl (J.M.B); gespina@bioscience.cl (G.E)

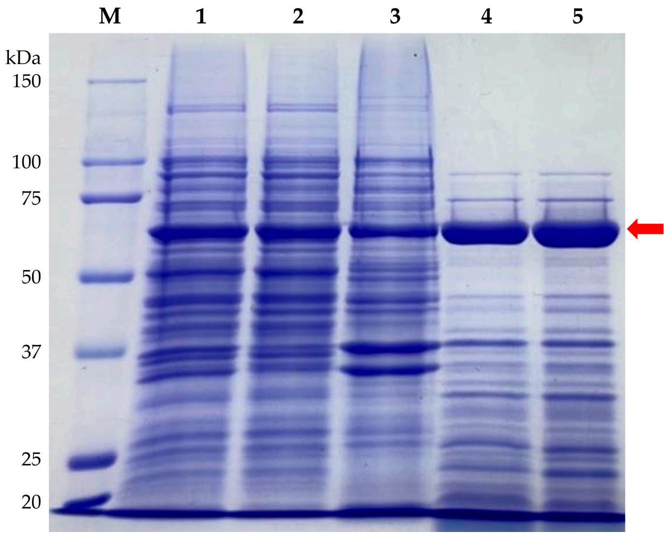

Figure S1. Electrophoretic analysis of the heterologous overexpression and partial purification of FNTL. Lane M: Molecular weight marker (Biorad Precision Plus Protein™ Kaleidoscope™ prestained protein standard). Lane 1: *E. coli* BL21 lysate (total fraction); Lane 2: inclusion bodies solubilized with 8 M urea (insoluble fraction); Lane 3: cell-free extract (soluble fraction); Lane 4: partial purification by heat treatment at 85°C for 5 min. Lane 5: concentrated partially purified FNTL. The protein concentration loaded in each well is 15 µg and the band corresponding to FNTL is highlighted by a red arrow

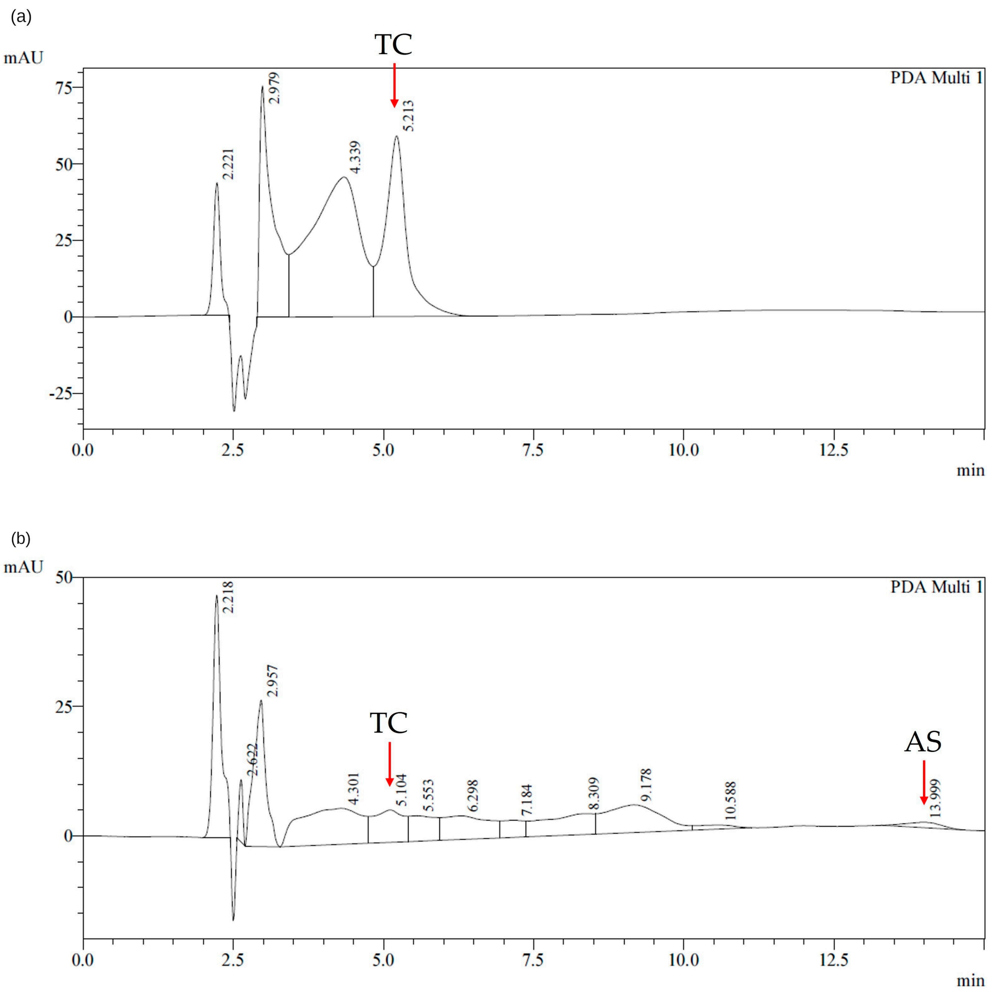

Figure S2. Chromatograms of tetracycline separation by reversed phase HPLC. **(a)** Chromatogram obtained from control tetracycline incubated at pH 6.0, 20°C for 24 h. **(b)** Chromatogram obtained from tetracycline incubated with FNTL and AS at pH 6.0, 20°C for 24 h.

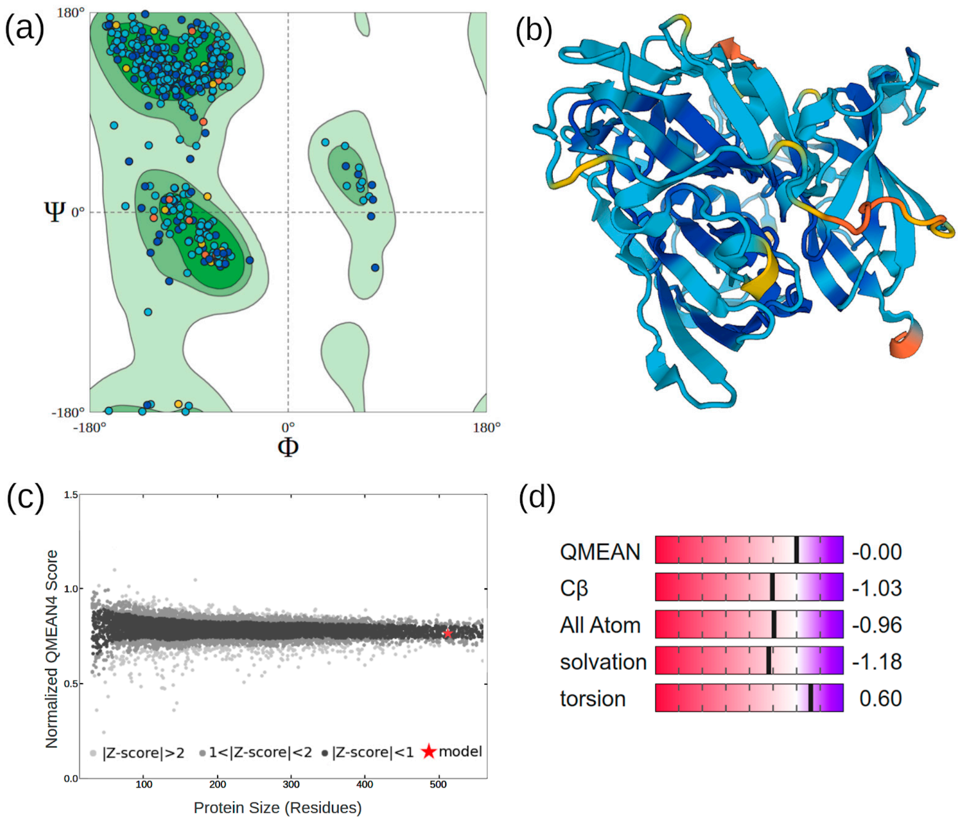

Figure S3. Quality assessment of the 3D model of FNTL inferred by homology with SWISS-MODEL. **(a)** Ramachandran plot of the conformational space of the three-dimensional model; **(b)** illustrating the distribution of phi (φ) and psi (ψ) angles for amino acid residues in energetically allowed and forbidden regions, and **(c)** comparison with non-redundant sets of PDB structures based on QMEAN4 scores. **(d)** Geometric evaluators of the model (Cβ interaction, all-atom interaction, solvation, torsion). Ramachandran plots were generated using RamachanDraw; Z-score-transformed QMEAN4 score plots were generated using the Swiss-Model server.

Table S1. Quality scores of FNTL three-dimensional models generated with SWISS-MODEL. ERRAT score: percentage of the protein residues whose error value falls below the 95% rejection cutoff; Verify 3D score: percentage of residues with averaged 3D-1D score ≥ 0.1; Prosa Z-score and QMEANDisCo scores represent overall model quality. MolProbity-score represents the crystallographic resolution at which the observed quality values would be expected. Model 1 and Model 2 correspond to the models built using as templates A0A6I7FGV2 and 1GSK, respectively.

Validation tool		Swiss-Model's FNTL models	
	Scoring range	Model 1	Model 2
**ERRAT (%)**	[0–100]	89.33	87.29
**Verify 3D (%)**	[0–100]	86.35	85.85
**Prosa**	[–]	-7.88	-7.98
**MolProbity (Å)**	[0–99]	1.03	1.40
**QMEANDisCo**	[0–1]	0.85	0.87
